# Cross-Sectional Survey of Childhood Acetabular Development in Japan

**DOI:** 10.31662/jmaj.2019-0035

**Published:** 2019-09-10

**Authors:** Yuta Tsukagoshi, Makoto Kamegaya, Hiroshi Kamada, Mitsuaki Morita, Yohei Tomaru, Shogo Nakagawa, Mio Kimura, Ryoko Takeuchi, Tomofumi Nishino, Masashi Yamazaki

**Affiliations:** 1 Department of Pediatric Orthopaedic Surgery, Ibaraki Children’s Hospital (Tsukuba Pediatric Orthopaedic Group), Mito, Japan; 2 Chiba Child & Adult Orthopaedic Clinic (Chiba Pediatric Orthopaedic Group), Chiba, Japan; 3 Department of Orthopaedic Surgery, University of Tsukuba (Tsukuba Pediatric Orthopaedic Group), Tsukuba, Japan; 4 Department of Orthopaedic Surgery, Ibaraki Prefectural University of Health Sciences (Tsukuba Pediatric Orthopaedic Group), Ami, Japan

**Keywords:** acetabular development, cross-sectional survey, developmental dysplasia of the hip, child, acetabular dysplasia

## Abstract

**Introduction:**

We aimed to clarify childhood acetabular development and to identify the incidence of children’s hip dysplasia in Japan using radiographs of the contralateral hip.

**Methods:**

We performed radiological cross-sectional evaluation of hip development in 211 patients (106 boys, 211 hips) in different age groups (age range: 3–9 years). We excluded patients who complained of bilateral coxalgia at the first visit or had received a diagnosis of acetabular dysplasia. We measured the acetabular index (AI), center-edge angle (CEA), and acetabular head index (AHI) in plain radiographs taken at the first visit.

**Results:**

A significant correlation was found between age and CEA in boys, but other parameters had no significant correlation. The mean AI values in boys and girls were 18 ± 3° and 20 ± 4° (p < 0.01), respectively, and the mean CEA values were 25 ± 5° and 24 ± 5° (p = 0.43), respectively. The mean AHI values in boys and girls were 83 ± 6% and 81 ± 7%, respectively (p < 0.01). Two of the 120 children (66 boys and 54 girls) aged ≥6 years old had a hip CEA < 15°; both were girls.

**Conclusions:**

We found decreased acetabular development in girls, and 4% (2/54) of girls without any history of dislocation belonged to Severin’s group III. Acetabular dysplasia was observed more frequently in children from Japanese than in those from other countries. Girls with less than two standard deviations in hip dysplasia indices had an AI of 28°, an AHI of 67%, and a CEA of 14°. These reference values may be useful as prognostic indicators for hip dysplasia and OA in adulthood.

## Introduction

Approximately 75% of Japanese patients with osteoarthritis of the hip (OA) are women with primary acetabular dysplasia, most of whom have no history of hip dislocation ^(1)^. Treatment during childhood is important to prevent the progression from acetabular dysplasia to OA; however, the optimal treatment for primary acetabular dysplasia in children is unclear because of a lack of epidemiological information on normal acetabular development in childhood. Previous studies ^(2), (3), (4)^ have analyzed aggregated radiographic data, but these evaluations were performed in normal hips, which would not reveal the prevalence of underlying primary acetabular dysplasia. In Japan, there are more adults with acetabular dysplasia than in other countries; ^(5), (6)^ thus, a unique reference index for OA has been suggested from a survey of hip radiograms in Japanese adults ^(5), (7), (8), (9)^. Therefore, we sought to clarify normal acetabular development and morphology in children by evaluating radiographs of contralateral side hips in patients with transient hip synovitis ^(10)^ and to determine factors related to the progression to hip dysplasia, which will consequently result in osteoarthritis in adulthood in this population.

## Materials and Methods

We evaluated 211 hips (211 patients, 106 boys, age range: 3–9 years) diagnosed with transient hip synovitis. Plain hip radiographs were taken at the first visit. Patients with bilateral hip symptoms or history of developmental hip dislocation were excluded. Radiographs were obtained in an anteroposterior view with the patient in the supine position with both hips in neutral rotation. Neutral rotation was determined using the Tönnis definition, which defines a ratio of the widths of the obturator foramen between 0.56 and 1.8 ^(11)^, with lower limbs fully extended and parallel to each other. We measured the acetabular index (AI), center-edge angle (CEA), and acetabular head index (AHI) ^(12)^ of the intact hip in all patients. CEA was measured with reference to the point of lateral edge of acetabular, called classical CEA. Children aged 6–9 years old were classified according to Severin’s classification ^(13)^. Furthermore, we compared parameters of the same side in 43 radiographs (43 patients) at the first visit with those at a later visit, which were taken to monitor for signs of mild symptomatic Legg–Calvé–Perthes disease at every 1 to 3 months after the first visit until hip pain resolved, to validate the adequacy of radiography for identifying transient hip synovitis.

To compare outcomes between boys and girls, an independent sample’s Student’s *t*-test was performed. To determine the correlation with age, Pearson's correlation coefficient was used. All analyses were performed using SPSS 24.0J (IBM, Chicago, IL, USA). Statistical significance was set at p < 0.01. This research was approved by the institutional review boards of the authors’ affiliated institutions.

We did not have a written consent because we used epidemiological surveys based on radiographs of other common diseases. The retrospective review of medical records and the radiographs received the approval of the institutional review board in Chiba Child and Adult Orthopaedic Clinic (#18-001).

## Results

**Figure 1.**
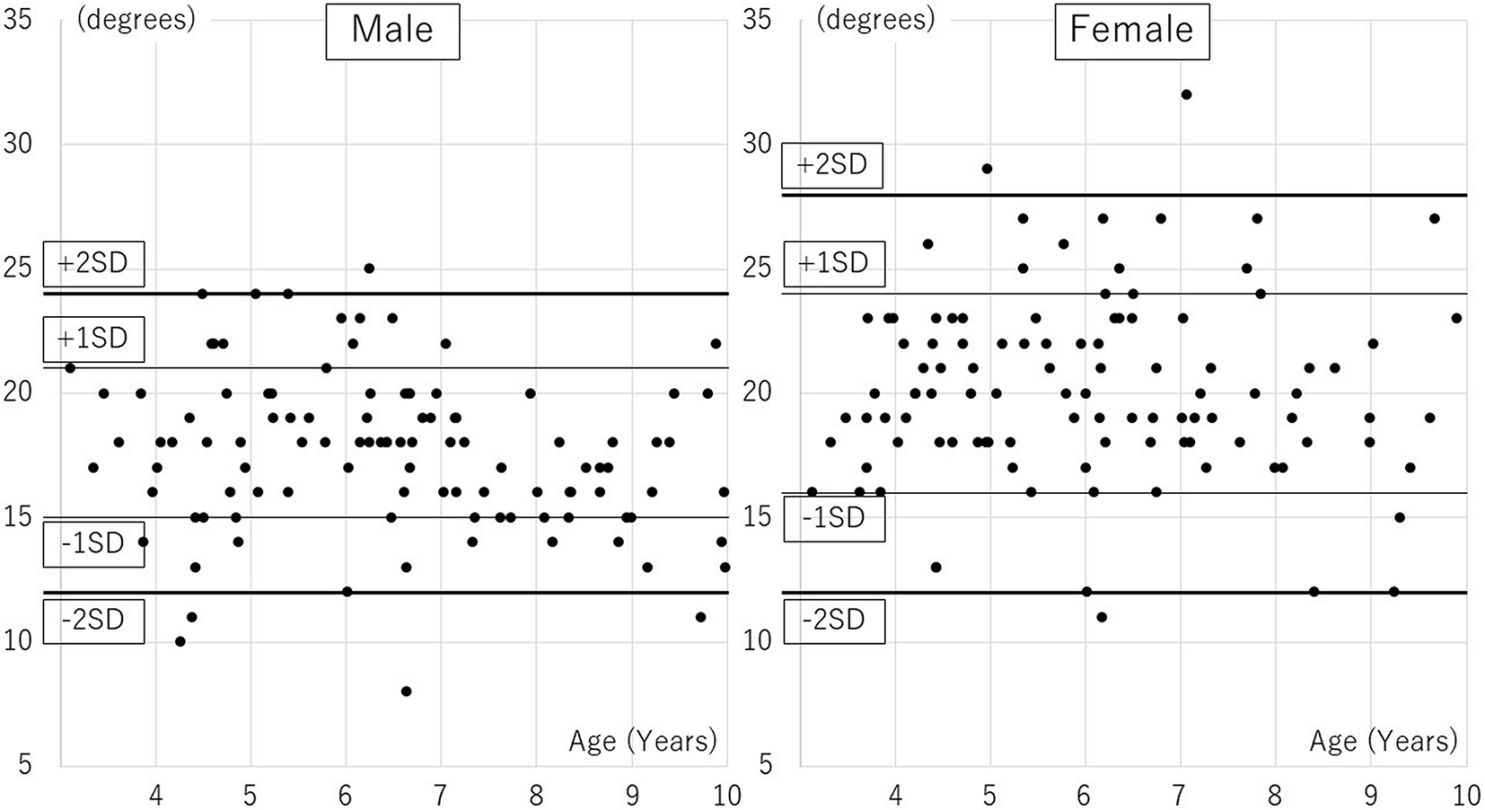
Acetabular index dispersion diagram.

**Figure 2.**
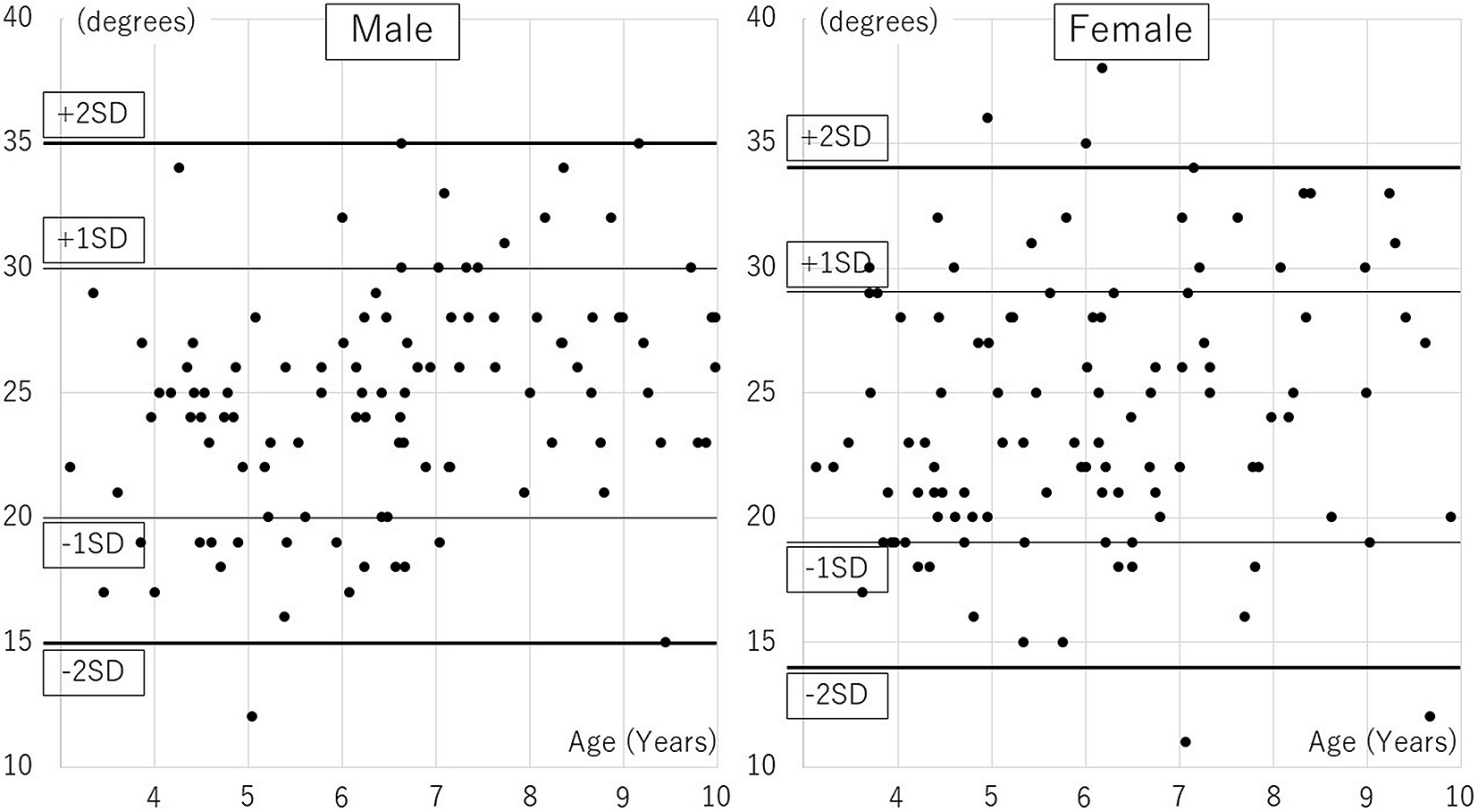
Center-edge angle dispersion diagram.

The age distribution of participants was followed: 7 boys and 12 girls aged 3, 20, and 24 aged 4, 13, and 15 aged 5, 26, and 22 aged 6, 14, and 16 aged 7, 15, and 9 aged 8, 11, and 7 aged 9. Distributions of AI, CEA, and AHI are presented in Table 1, 2 and 3, Figure 1, 2, and 3. A significant correlation with age was observed only in CEA for boys (Table 4).

**Table 1.** Acetabular Index by Age and Gender.

		Total							Boys							Girls					
Age (years)		Avg		SD	Min		Max		Avg		SD	Min		Max		Avg		SD	Min		Max
3		17	±	3	(14	–	23)		18	±	3	(14	–	21)		19	±	3	(16	–	23)
4		19	±	4	(10	–	29)		17	±	4	(10	–	24)		20	±	3	(13	–	29)
5		21	±	3	(16	–	27)		20	±	3	(16	–	24)		21	±	3	(16	–	27)
6		19	±	4	(8	–	27)		18	±	4	(8	–	25)		20	±	4	(11	–	27)
7		19	±	4	(14	–	32)		17	±	2	(14	–	22)		21	±	4	(17	–	32)
8		17	±	2	(12	–	21)		16	±	1	(14	–	18)		18	±	3	(12	–	21)
9		18	±	4	(11	–	27)		16	±	3	(11	–	22)		19	±	5	(12	–	27)
All		19	±	4	(8	–	32)		18	±	3	(8	–	25)		20	±	4	(11	–	32)

Avg: average; SD: standard deviation; Min: Minimum; Max: Maximum.

**Table 2.** Center-edge Angle by Age and Gender.

		Total								Boys								Girls						
Age (years)		Avg		SD		Min		Max		Avg		SD		Min		Max		Avg		SD		Min		Max
3		23	±	4	°	(17	–	30)		23	±	4	°	(17	–	29)		23	±	4	°	(17	–	30)
4		23	±	4	°	(16	–	36)		24	±	4	°	(17	–	34)		23	±	5	°	(16	–	36)
5		23	±	5	°	(12	–	32)		21	±	4	°	(12	–	28)		24	±	5	°	(15	–	32)
6		24	±	5	°	(17	–	38)		24	±	5	°	(17	–	35)		24	±	5	°	(18	–	38)
7		26	±	5	°	(11	–	34)		27	±	4	°	(19	–	33)		25	±	6	°	(11	–	34)
8		27	±	4	°	(20	–	34)		27	±	4	°	(21	–	34)		28	±	4	°	(20	–	33)
9		25	±	6	°	(12	–	35)		26	±	5	°	(15	–	35)		24	±	8	°	(12	–	33)
All		24	±	5	°	(11	–	38)		25	±	5	°	(12	–	35)		24	±	5	°	(11	–	38)

Avg: average; SD: standard deviation; Min: Minimum; Max: Maximum.

**Table 3.** Acetabular Head Index by Age and Gender.

		Total							Boys							Girls					
Age (years)		Avg		SD	Min		Max		Avg		SD	Min		Max		Avg		SD	Min		Max
3		83	±	5	(75	–	91)		86	±	5	(76	–	91)		82	±	4	(75	–	88)
4		84	±	6	(71	–	96)		86	±	5	(76	–	93)		83	±	7	(71	–	96)
5		79	±	8	(65	–	94)		78	±	6	(67	–	89)		81	±	8	(65	–	94)
6		82	±	6	(69	–	95)		83	±	5	(75	–	95)		80	±	6	(69	–	93)
7		80	±	7	(66	–	94)		84	±	6	(76	–	94)		77	±	7	(66	–	86)
8		82	±	5	(74	–	91)		83	±	4	(76	–	89)		80	±	5	(74	–	91)
9		81	±	8	(63	–	92)		82	±	6	(68	–	89)		81	±	10	(63	–	92)
All		82	±	6	(63	–	96)		83	±	6	(67	–	95)		81	±	7	(63	–	96)

Avg: average; SD: standard deviation; Min: minimum; Max: maximum.

**Figure 3.**
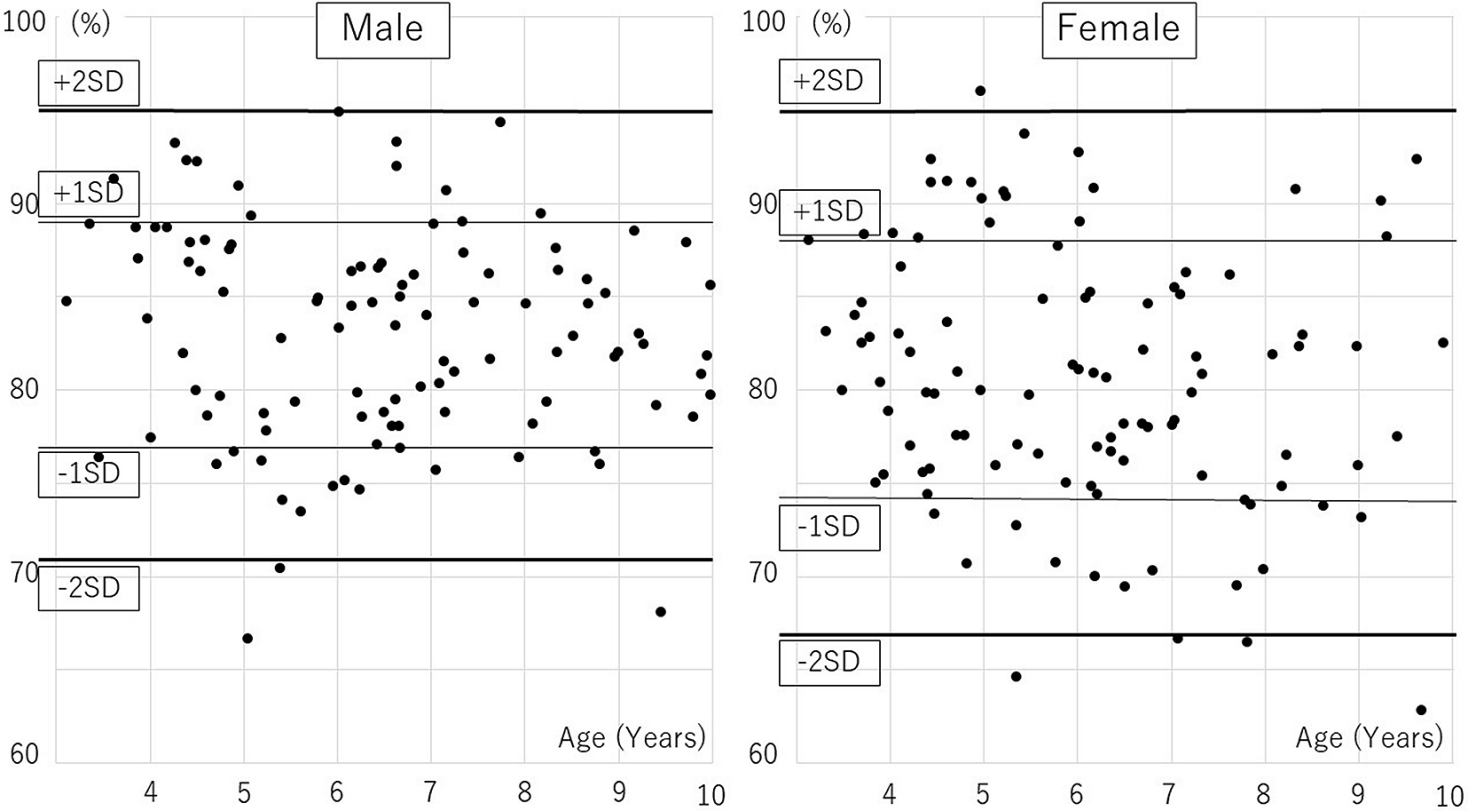
Acetabular head index dispersion diagram.

**Table 4.** Correlation between Gender and Acetabular Parameters.

		AI	CEA	AHI
**Boys**	Coefficient of correlation	−0.205	0.306	−0.118
	*p*-value	0.035	0.001*	0.229
**Girls**	Coefficient of correlation	−0.003	0.150	−0.192
	*p*-value	0.972	0.128	0.050

*statistically significant; AI: acetabular index; CEA: center-edge angle; AHI: acetabular head index

The mean AI values for boys and girls were 18 ± 3° and 20 ± 4°, respectively, (p < 0.01), and the mean CEA values were 25 ± 5° and 24 ± 5°, respectively (p = 0.43). The mean AHI values for boys and girls were 83 ± 6% and 81 ± 7%, respectively (p < 0.01). There were significant differences between boys and girls for AI and AHI. In Severin’s classification, 105 hips of the 120 hips (66 boys, 54 girls) aged ≥6 years had a hip CE angle ≥20°; they were categorized into Severin group Ia. Thirteen hips had a hip CE angle of 15°–20° (group Ib), and two had a hip CE angle <15° (group III). Both group-III children were girls aged 7 and 9 years. Differences in measurements between the first visit and the visit after hip pain resolution were as follows: AI, 0 ± 2°; CEA, −1 ± 2°; AHI, +1 ± 3%. These values were not significantly different; thus, radiographs in the contralateral side of unilateral transient hip synovitis were similar to those taken at the first visit.

## Discussion

We found a difference in AI and AHI between young boys and girls and that dysplasia was more prevalent in girls. Our findings were similar to those in previous reports ^(2), (11)^. Developmental hip dislocation is observed more often in girls; however, our results indicated that developmental dysplasia without dislocation was also more prevalent in girls.

In this study, there was no significant correlation between acetabular development and age in girls aged 3–9 years. A cross-sectional survey ^(3), (4)^ of bony AI with magnetic resonance imaging indicated that bony AI improved by 4 years of age; furthermore, there was no remarkable development between 5 and 8 years of age, but improvement was seen in children over 9 years of age. Participants in this study were 3–9 years old, and they may not have any bony acetabular development during this period based on MRI studies ^(3), (4)^. However, a significant correlation of CEA with age was observed for boys in our study.

Yamamuro et al. ^(14)^ surveyed hip radiographs in Japanese children aged < 4 years and reported mean AI values of 19 ± 3° in boys and 21 ± 3° in girls aged 3–4 years old, which were similar to our results. Contrarily, Tönnis ^(11)^ reported mean AI values in children aged 3–5 years of 16 ± 4° in boys and 17 ± 4° in girls, which differed from the results of other surveys in Japan. Moreover, Severin ^(13)^ surveyed 136 hips (68 patients) radiographically to establish a normal acetabular morphology in children aged 6–13 years and reported that a CEA of 15°–40° was observed in normal children; the author defined hips with a CEA of 15°–20°, as observed in approximately 2% of the participants, as group Ib, also called *uncertain cases*. In the 120 children aged 6–9 years old surveyed in the current study, 13 children (11%) were classified into Severin group Ib. The fact that almost all patients with OA due to acetabular dysplasia without history of developmental dysplasia of the hip (DDH) in Japan could be found in group Ib, the so-called uncertain cases. In Severin’s report, all children categorized into group III (CEA < 15°) had a history of hip dislocation. However, in the current study, we surveyed children without a history of hip dislocation, and two children (2%) were categorized into Severin group III; thus, it seems that pathological acetabular dysplasia is latent in Japanese children. Both group-III children were girls, 7 and 9 years old; therefore, 4% of girls aged 6–9 years were classified as group III in this study. Acetabular dysplasia was observed more frequently in Japanese children than in other countries (Table 5).

**Table 5.** Comparison of Childhood Acetabular Development Indices with Data from Other Countries.

Measurement	Nation	Subjects	Age (years)		Result	
CEA				<15°	15°–19°	20°–40°
	Sweden^(13)^	Boys and girls	6–13	0%	2%	98%
	Japan*	Girls	6–9	4%	13%	83%
AI				≥24°	20°–23°	<19°
	Germany^(11)^	Girls	5–7	2%	16%	82%
	Japan*	Girls	5–7	23%	35%	42%

*Current study; CEA: center-edge angle; AI: acetabular index

Upon physical examination of 2,975 adult citizens (mean age: 70.2 [23–94] years) living in selected cities in Japan ^(15)^, the rate of coxalgia was found to be 0.58% (6/1,043) in men and 2.56% (49/1,932) in women, while the rate of Kellgren–Lawrence ^(16)^ grade 2 or above OA was 0.29% (3/1,043) in men and 0.99% (19/1,932) in women; therefore, the prevalence of OA in women was much higher. In a survey of 485 Japanese (52 men, 433 women) who visited an outpatient clinic for coxalgia and were diagnosed with OA ^(1)^, 72% (349/485) were women with hip dysplasia, but most had no history of DDH in childhood. By contrast, among the 28% (136/485) diagnosed with DDH in childhood, only 4% (19/485) underwent surgery in childhood ^(1)^. In fact, based on this epidemiological data, it can be concluded that nearly one-fourth of the women with post-DDH OA in the current study might have had been spared from OA changes with supplemental surgery in childhood.

Albinana et al. ^(17)^ evaluated the outcome in 72 hips of 58 patients with DDH who underwent hip reduction by 16 months of age and had no history of secondary surgery; they reported that 80% of hips with an AI 28° or worse 4 years after reduction were found in patients with a mean age of 5.5 years, who were subsequently classified into Severin group III or IV. In the current survey, 28° represented two standard deviations (SDs) of AI in girls.

Terjesen ^(18)^ evaluated the outcome of 23 hips with DDH (dislocation or subluxation) and CEA < 20° from age 8–10 years to over 45 years and reported that 22% (4/18) of those with an AHI of ≥67% and 80% (4/5) of those with AHI < 67% subsequently progressed to OA. In the current survey, the percentage of girls with −2 SDs of AHI was 67%.

Some previous reports ^(19), (20)^ confirmed that a CEA < 20° was a risk factor for OA in adult hips; however, other reports ^(21), (22)^ contradict that cut-off. In a survey of 71 patients with DDH (dislocation or subluxation) followed up over 50 years, researchers suggested that a mean CEA of 17.5 ± 1.3° among those aged 8–10 years old indicate the likelihood of them eventually developing OA ^(23)^. Moreover, in a survey of adult patients with acetabular dysplasia with or without a history of dislocation followed over 10 years, a mean CEA of 12.9 ± 9.1° was proposed as necessary to avoid OA development ^(24)^.

McWilliams et al. defined a cut-off value of −1.96 SD of CEA by normal hip radiography of 1,108 patients aged >45 years and reported that dysplastic hips with a CEA of −1.96 SD or less had an 8-fold higher risk of developing OA ^(25)^. Thus, as AI, CEA, and AHI values of −2 SD from the normal reference values in children are defined as clinically important in hip dysplasia and consequent OA, these values might be useful as prognostic indicators for these pathologies in adulthood.

This study has several limitations. First, we could not perform the LMS method (normalization of reference values using skewness (L), the median (M), and the coefficient of variation (S)) ^(26)^ for adequately constructing normalized growth standards due to the small number of subjects included in this study. Second, we were unable to evaluate older children, because we included only those patients with transient synovitis of the hip, which is a common disease in children under 10 years of age.

In conclusion, this cross-sectional survey revealed that acetabular development is different in young boys and girls, and that dysplasia was more prevalent in girls in this age group. We clarified acetabular development radiographically in Japanese children and found that 4% of girls without any history of dislocation belonged to Severin’s group III. Less than 2 SD for hip dysplasia in girls corresponded to an AI of 28°, an AHI of 67%, and a CEA of 14°. These reference values might be useful as prognostic indicators for hip dysplasia and OA in adulthood.

## Article Information

### Conflicts of Interest

None

### Author Contributions

**Yuta Tsukagoshi**: Conception and design of the study, analysis and interpretation of data, collection and assembly of data, drafting of the article, critical revision of the article for important intellectual content.

**Makoto Kamegaya**: Conception and design of the study, analysis and interpretation of data, collection and assembly of data, drafting of the article, 

**Hiroshi Kamada**: Conception and design of the study, conception and design of the study.

**Mitsuaki Morita**: Conception and design of the study, collection and assembly of data.

**Yohei Tomaru**: Conception and design of the study, collection and assembly of data.

**Shogo Nakagawa**: Conception and design of the study.

**Ryoko Takeuchi**: Conception and design of the study.

**Mio Onishi**: Conception and design of the study.

**Tomofumi Nishino**: Conception and design of the study.

**Masashi Yamazaki**: Conception and design of the study, final approval of the article.

### Approval by Institutional Review Board (IRB)

Chiba Child & Adult Orthopaedic Clinic #18-001
